# Publisher Correction: Chloroquine modulates antitumor immune response by resetting tumor-associated macrophages toward M1 phenotype

**DOI:** 10.1038/s41467-018-04169-w

**Published:** 2018-05-01

**Authors:** Degao Chen, Jing Xie, Roland Fiskesund, Wenqian Dong, Xiaoyu Liang, Jiadi Lv, Xun Jin, Jinyan Liu, Siqi Mo, Tianzhen Zhang, Feiran Cheng, Yabo Zhou, Huafeng Zhang, Ke Tang, Jingwei Ma, Yuying Liu, Bo Huang

**Affiliations:** 1 Department of Immunology & National Key Laboratory of Medical Molecular Biology, Institute of Basic Medical Sciences, Chinese Academy of Medical Sciences, 100005 Beijing, China; 2 Department of Biochemistry & Molecular Biology, Tongji Medical College, Huazhong University of Science & Technology, 430030 Wuhan, China; 3 Clinical Immunology Center, Chinese Academy of Medical Sciences, 100005 Beijing, China

**Correction to**: *Nature Communications* 10.1038/s41467-018-03225-9, published online 28 February 2018.

In the originally published version of this Article, images in Fig. 5n were inadvertently replaced with duplicates of images in Fig. 5o during the production process. This has now been corrected in both the PDF and HTML versions of the Article.

