# Supplementary material for: Chloroquine modulates antitumor immune response by resetting tumor-associated macrophages toward M1 phenotype
Source: Nat Commun. 2018 Feb 28;9:873. doi: 10.1038/s41467-018-03225-9 (PMC5830447; doi:10.1038/s41467-018-03225-9)
Supplement: Supplementary file 1 — Supplementary Information [file 41467_2018_3225_MOESM1_ESM.pdf]

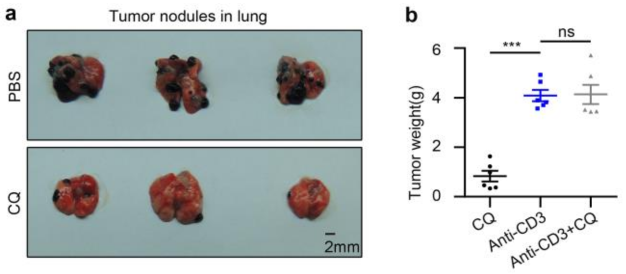

Supplementary Figure 1. Related to Figure 1.

a, Three representative lungs each from PBS and CQ treatment groups were photographed. Each black spot is a macroscopic B16 melanoma nodule. Scale bar, 2mm.

b, C57BL/6 mice (n=6) bearing subcutaneous B16 melanoma were i.p. treated with anti-CD3 neutralizing antibody and/or CQ. After two weeks, the mice were sacrificed and the tumor weight was measured. Data shown are representative of three independent experiments and error bars represent mean ± s.e.m.. *, P <0.05; **, P <0.01; ***, P <0.001; ns, not statistically significant (Student's *t*-test).

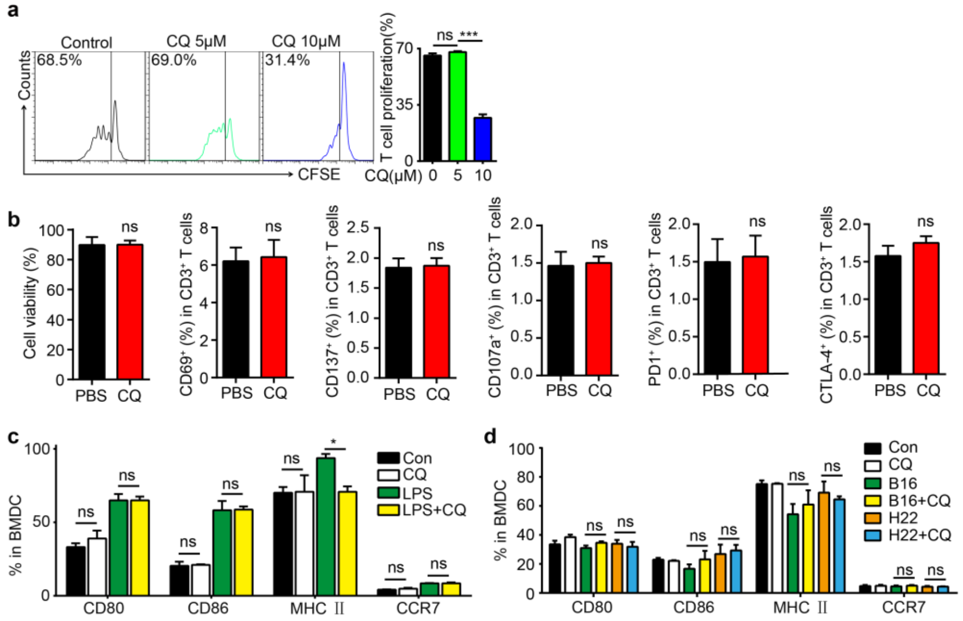

Supplementary Figure 2. Related to Figure 2.

a, Splenic CD3 T cells were labelled with carboxyfluorescein succinimidyl ester (CFSE) and then stimulated with 0μM, 5μM, 10μM CQ in the presence of CD3/CD28 beads and IL-2 for 72 hours. Cell proliferation was measured as shown by representative histograms of CFSE (left panel) and quantification of T cell proliferation (right panel) (n=3). **b,** The T cell viability (Annexin V and PI negative cells), T cell activation (CD69, CD137), lysosome status (Lamp1) as well as T cell anergy (PD1, CTLA-4) of splenic CD3 T cells with or without CQ treatment were analyzed (n=3). **c,** BMDCs were treated with 10μM CQ or PBS for 24 hours in the presence of LPS or not, the expression of CD80, CD86, MHCII, CCR7 were analyzed (n=3). **d,** B16 or H22 cells with CQ treatment or not were co-cultured with BMDCs (10:1), the expression of CD80, CD86, MHCII, CCR7 were analyzed (n=3). Data shown are representative of three independent experiments and error bars represent mean ± s.e.m.. *, P <0.05; **, P <0.01; ***, P <0.001; ns, not statistically significant (Student's t-test).

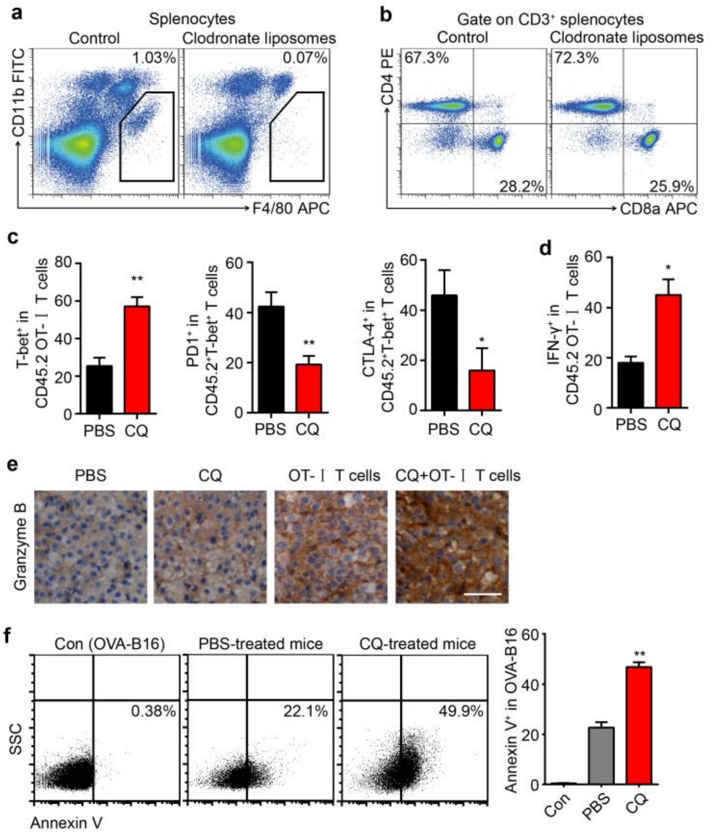

Supplementary Figure 3. Related to Figure 2.

a, The number of F4/80^{hi} and CD11b$^{lo/int}$ cells were analyzed by flow cytometry after two days with or without 200μL clodronate liposomes treatment in the spleen of C57BL/6 mice. **b,** The number of splenic CD4^{+} and CD8^{+} events within the CD3^{+} T cell gate were quantified by flow cytometry after two days with or without 200μL clodronate liposomes treatment in C57BL/6 mice. **c,d,e** OVA-B16 melanoma-bearing CD45.1^{+} C57BL/6 mice were treated with or without CQ, concomitant with adoptive transfer of OVA-specific CD8^{+} T cells from CD45.2^{+} OT-I transgenic mice. The expression of T-bet in CD45.2^{+} OT-I T cells and PD1, CTLA-4, IFN-γ in CD45.2^{+}T-bet^{+} T cells were measured by flow cytometry (**c,d, n=3**); the expression of granzyme B in tumor tissues were analyzed by immunohistochemical staining (**e**). Scale bar,

50μm. **f,** Same experimental conditions as **c,d,e**, CD45.2^{+} OT-I T cells were sorted from PBS or CQ-treated tumor tissues and co-cultured with OVA-B16 as a ratio of 30:1. Representative flow cytometric analysis (left) and quantification (right) showing Annexin V^{+} in OVA-B16 cells (n=3). Data shown are representative of three independent experiments and error bars represent mean ± s.e.m.. *, P <0.05; **, P <0.01; ***, P <0.001; ns, not statistically significant (Student's *t*-test).

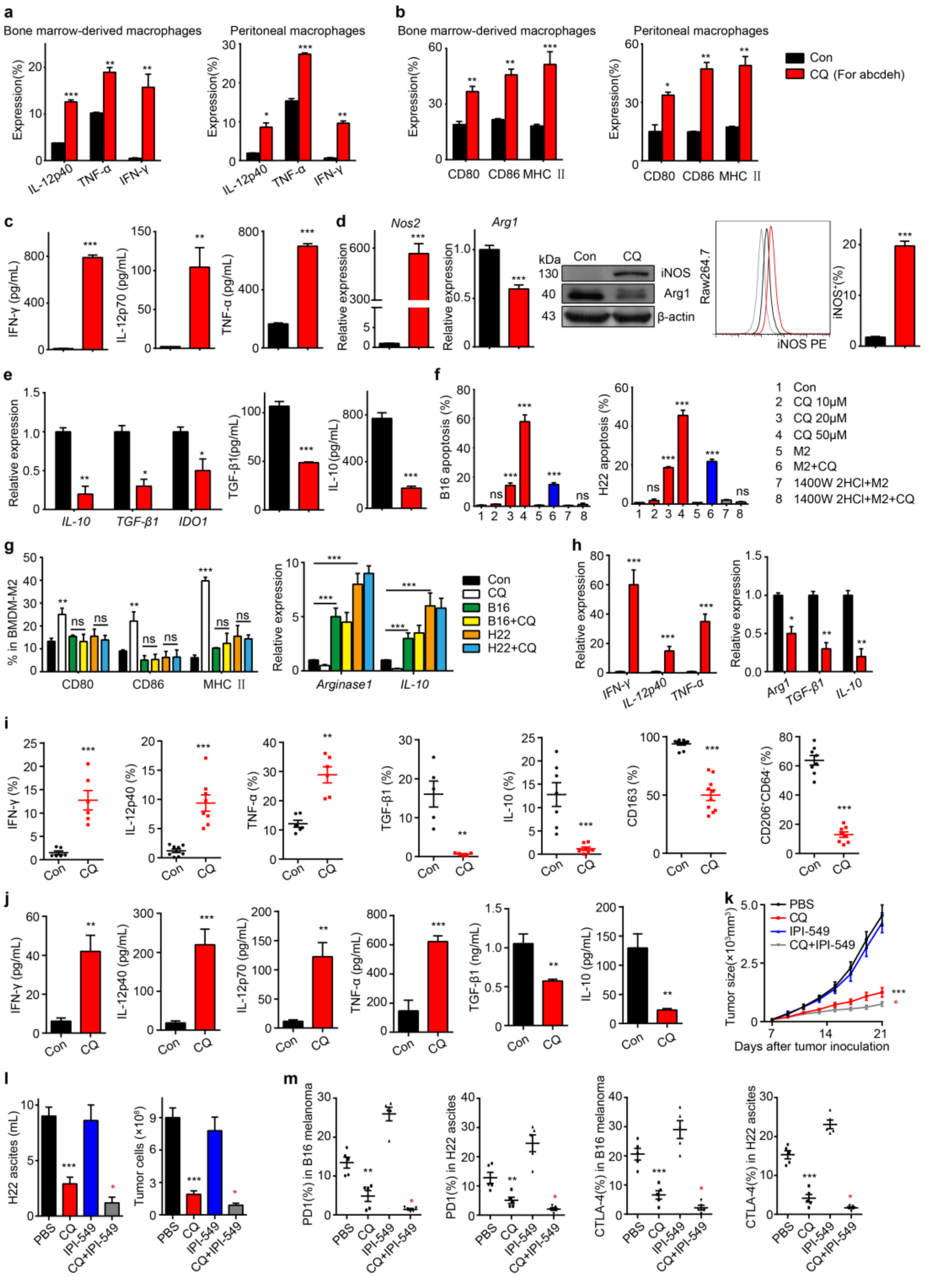

Supplementary Figure 4. Related to Figure 3.

a, Quantification of IL-12p40, TNF-α and IFN-γ in BMDMs (left) and peritoneal macrophages (right) with or without CQ treatment (n=3). **b,** Quantification of CD80, CD86 and MHC II in BMDMs (left) and peritoneal macrophages (right) with or without

CQ treatment (n=3). **c,** Quantification of IFN-γ, IL-12p70 and TNF-α in the supernatants of BMDM-M2s with or without CQ treatment were measured by ELISA (n=3). **d,** The mRNA expression of *NOS2* and *Arg1* in IL-4 conditioned Raw264.7 macrophages with or without CQ treatment were analyzed by real-time qPCR (left, n=3); the expression of iNOS, Arg1 and β-actin in IL-4 conditioned Raw264.7 macrophages with or without CQ treatment were analyzed by western blot (center); representative flow cytometric analysis and quantification of iNOS in IL-4 conditioned Raw264.7 macrophages with or without CQ treatment are shown (right, n=3). **e,** The mRNA expression of *IL-10, TGF-β1, IDO1* in BMDM-M2 cells with or without CQ treatment were analyzed by real-time qPCR (n=3); the production of TGF-β1, IL-10 in the supernatants of BMDM-M2s with or without CQ treatment were measured by ELISA (n=3). **f,** B16 (left) and H22 (right) cells were treated with 10μM, 20μM, 50μM CQ; conditional medium of BMDM-M2s with or without CQ treatment; conditional medium of BMDM-M2s with or without CQ and/or 1400W 2HCl treatment. The cell apoptosis was analyzed by Annexin V and PI staining (n=3). **g,** B16 or H22 cells with CQ treatment or not were co-cultured with BMDM-M2s (10:1), the expression of CD80, CD86, MHCII were analyzed by flow cytometry (n=3), the mRNA expression of *Arg1* and *IL-10* were analyzed by real-time qPCR (n=3). **h,i,j** Human M2-like macrophages were treated with CQ or not, the mRNA expression of *IFN-γ, IL-12p40, TNF-α, Arg1, TGF-β1, IL-10* were analyzed by real-time qPCR (**h**, n=3); the IFN-γ (n=6), IL-12p40 (n=8), TNF-α (n=6), TGF-β1 (n=5), IL-10 (n=7), CD163 (n=10), $CD206^{+}CD64^{-}$ (n=8) were analyzed by flow cytometry (**i**); the IFN-γ, IL-12p40, IL-12p70, TNF-α, TGF-β1,

IL-10 in the supernatants were measured by ELISA (**j**, n=3). **k,l,m,** B16 or H22-bearing mice were treated with PBS, CQ, IPI-549 and CQ+IPI-549. The B16 growth was followed (**k,** n=10); the H22 ascites and tumor cell number were measured (**l,** n=8); the PD1 and CTLA-4 expression in $CD3^{+}CD8^{+}$ tumor infiltration T cells were analyzed (**m,** n=5). Black asterisk, CQ group compared with PBS group; red asterisk, CQ group compared with CQ+IPI-549 group. Data shown are representative of three independent experiments and error bars represent mean ± s.e.m.. *, P <0.05; **, P <0.01; ***, P <0.001; ns, not statistically significant (Student's *t*-test).

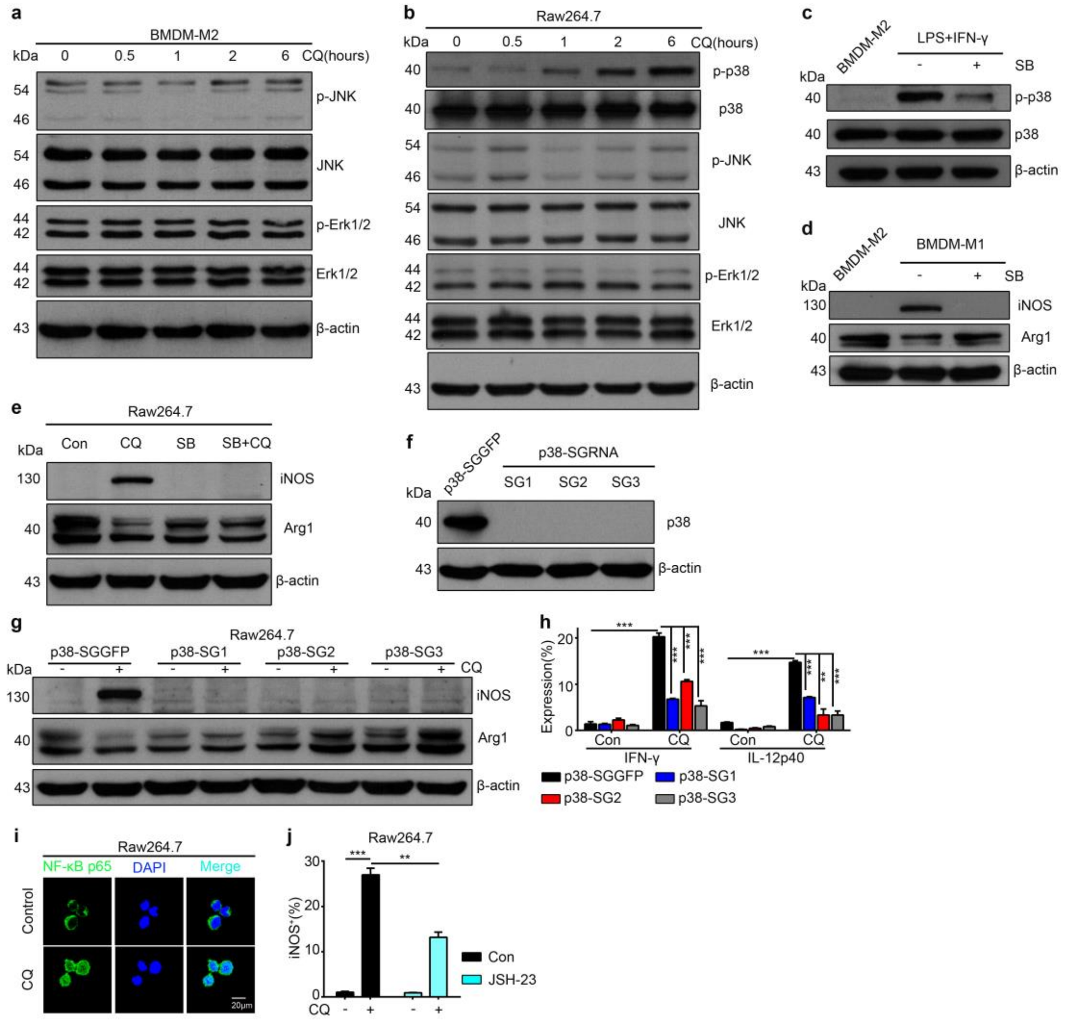

Supplementary Figure S5. Related to Figure 4.

a, The expression of p-JNK, total JNK, p-Erk1/2, total Erk1/2 and β-actin in BMDM-M2s treated with CQ at indicated time points were analyzed by western blot. **b,** The expression of p-p38, total p38, p-JNK, total JNK, p-Erk1/2, total Erk1/2 and β-actin in IL-4 conditioned Raw264.7 macrophages treated with CQ at indicated time points were analyzed by western blot. **c,** The expression of p-p38, total p38 and β-actin were analyzed by western blot in BMDM-M2s as well as BMDMs that had been pretreated for 2 hours with 10μM SB203580 or not and then stimulated for 2 additional hours with LPS and IFN-γ or PBS. **d,** The expression of iNOS, Arg1 and β-actin were analyzed by

western blot in BMDM-M2s as well as BMDM-M1s (BMDMs stimulated with LPS and IFN-γ for 24 hours) pretreated with or without SB203580 for 2 hours. **e,** The expression of iNOS, Arg1 and β-actin were analyzed by western blot in IL-4 conditioned Raw264.7 macrophages pretreated with 10μM SB203580 or not for 2 hours before addition of CQ or PBS. **f,** The expression of total p38 and β-actin were analyzed by western blot in Raw264.7 macrophages after p38-SGGFP and p38-SGRNA (SG1, SG2, SG3) manipulation by CRISPR/Cas9 technology. **g,** The expression of iNOS, Arg1 and β-actin were analyzed by western blott in p38-SGGFP, p38-SG1, p38-SG2 and p38-SG3 Raw264.7 macrophages with or without CQ treatment. **h,** IFN-γ and IL-12p40 production were analyzed by flow cytometry in p38-SGGFP, p38-SG1, p38-SG2 and p38-SG3 Raw264.7 macrophages with or without CQ treatment (n=3). **i,** The immunofluorescent staining of NF-κBp65 with or without CQ treatment in IL-4 conditioned Raw264.7 macrophages were visualized by confocal fluorescent microscopy. Green, NF-κBp65; blue, DAPI. Scale bar, 20μm. **j,** iNOS expression was quantified by flow cytometry in IL-4 conditioned Raw264.7 macrophages that had been pretreated with 10μM JSH-23 or not for 2 hours before addition of CQ or PBS. Data shown are representative of three independent experiments and error bars represent mean ± s.e.m.. *, P <0.05; **, P <0.01; ***, P <0.001; ns, not statistically significant (Student's *t*-test).

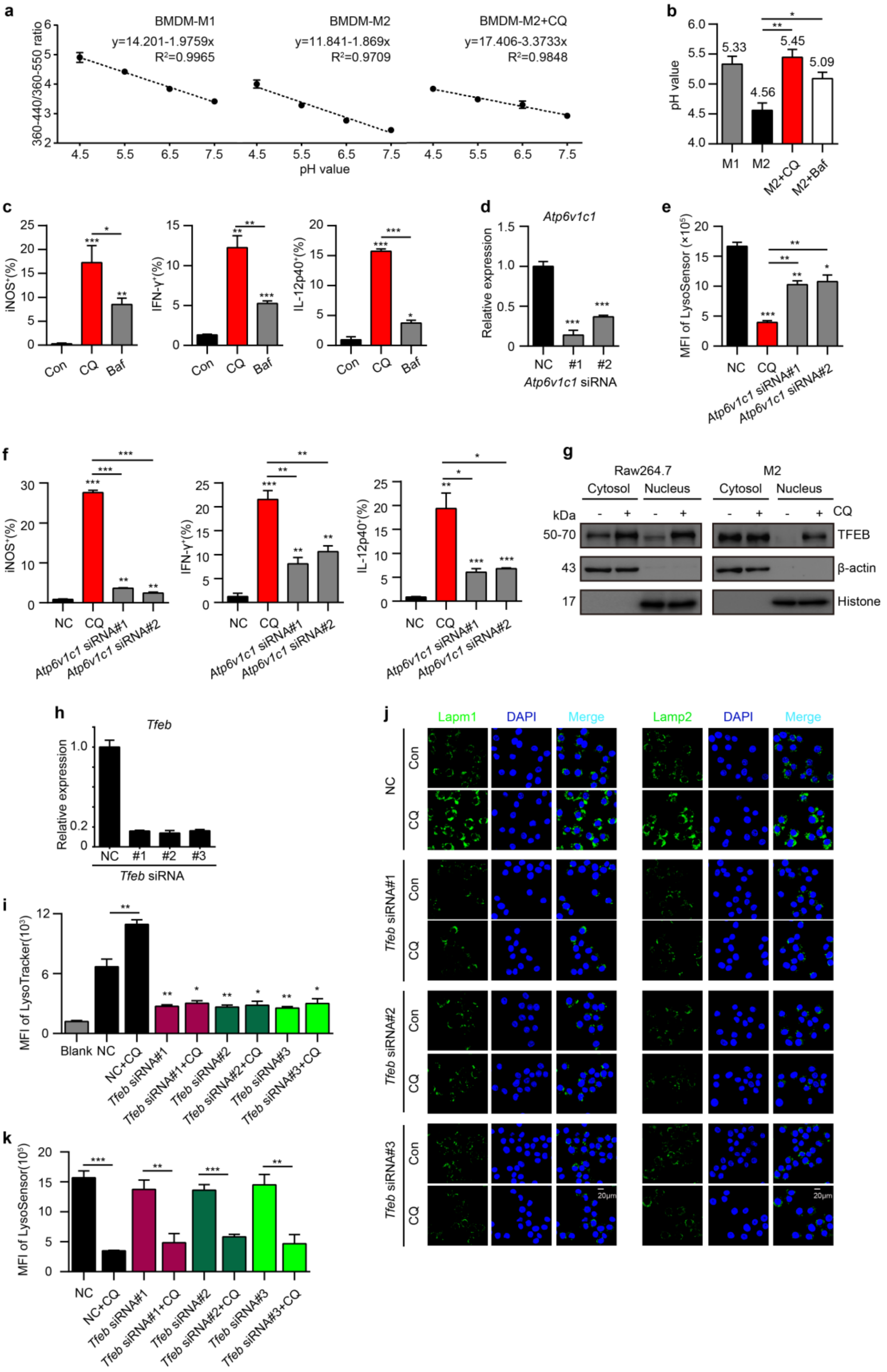

Supplementary Figure S6. Related to Figure 5.

a, The standard curves of lysosomal pH value in BMDM-M1s, BMDM-M2s as well as

in CQ-treated BMDM-M2s. **b,** The lysosomal pH value of BMDM-M1, BMDM-M2, CQ-treated and Baf-treated BMDM-M2 cells (n=3). **c,** The expression of iNOS, IFN-γ, IL-12p40 in CQ or Baf-treated BMDM-M2 cells were analyzed by flow cytometry (n=3). **d,** The mRNA expression of *Atp6v1c1* in Raw264.7 macrophages tranfected with control (NC) siRNA, *Atp6v1c1* siRNA#1 and #2 were analyzed by real-time qPCR (n=3). **e,** Flow cytometric quantification of the mean fluorescence intensity (MFI) as a marker for lysosomal pH with LysoSensor Green staining in CQ-treated or *Atp6v1c1* siRNA Raw264.7 cells (n=3). **f,** The expression of iNOS, IFN-γ, IL-12p40 in CQ or *Atp6v1c1* siRNA Raw264.7 macrophages were analyzed by flow cytometry (n=3). **g,** Cytoplasmic and nuclear fractionation analysis of TFEB in Raw264.7 and BMDM-M2s with or without CQ treatment by western blot. **h,** The mRNA expression of *Tfeb* in Raw264.7 macrophages tranfected with control (NC) siRNA, *Tfeb* siRNA#1, #2 and #3 were analyzed by real-time qPCR (n=3). **i,** Flow cytometric quantification of lysosome mass with LysoTracker Green staining in NC and *Tfeb* siRNA Raw264.7 cells with or without CQ treatment (n=3). **j,** Immunofluorescent analysis of Lamp1 (left panel) and Lamp2 (right panel) in NC and *Tfeb* siRNA Raw264.7 cells with or without CQ treatment were observed by confocal fluorescent microscope. Green, Lamp1 or Lamp2; Blue, DAPI; Scale bar, 20μm. **k,** Flow cytometric quantification of the MFI as lysosomal pH with LysoSensor Green staining in NC and *Tfeb* siRNA Raw264.7 cells with or without CQ treatment (n=3). Data shown are representative of three independent experiments and error bars represent mean ± s.e.m.. *, P <0.05; **, P <0.01; ***, P <0.001; ns, not statistically significant (Student's *t*-test).

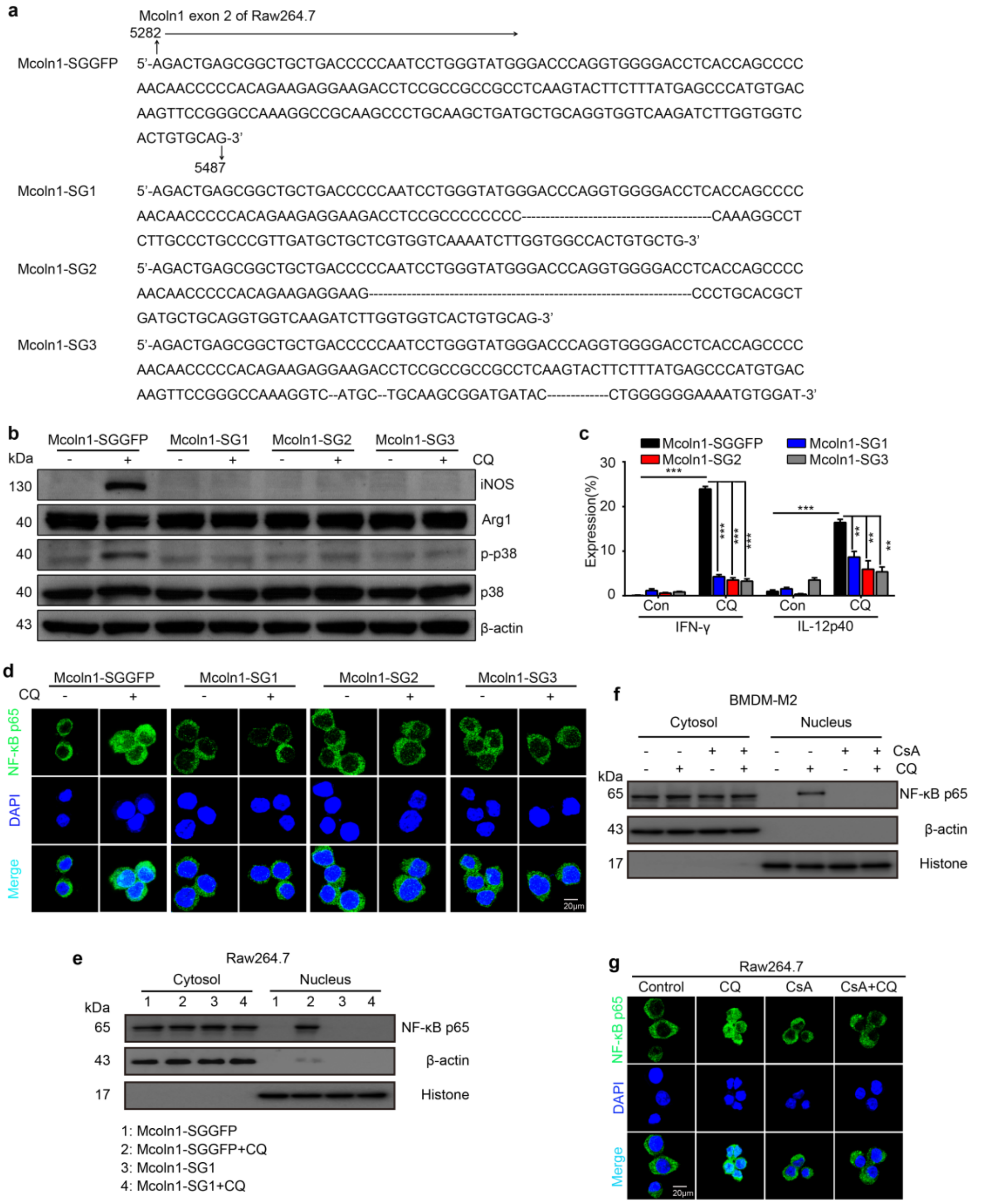

Supplementary Figure S7. Related to Figure 5.

a, The Mcoln1-exon2 sequencing results in Raw264.7 macrophages after CRISPR/Cas9 manipulation using Mcoln1-SGGFP, Mcoln1-SG1, Mcoln1-SG2 and Mcoln1-SG3 plasmids. **b,** The expression of iNOS, Arg1, p-p38, total p38 and β-actin in Mcoln1-SGGFP, Mcoln1-SG1, Mcoln1-SG2 and Mcoln1-SG3 Raw264.7 macrophages with or without CQ treatment were analyzed by western blot. **c,**

Quantification of IFN-γ and IL-12p40 in Mcoln1-SGGFP, Mcoln1-SG1, Mcoln1-SG2 and Mcoln1-SG3 Raw264.7 macrophages with or without CQ treatment were analyzed by flow cytometry (n=3). **d,** Immunofluorescent staining of NF-κBp65 was visualized by confocal fluorescent microscopy in IL-4 conditioned Mcoln1-SGGFP, Mcoln1-SG1, Mcoln1-SG2 and Mcoln1-SG3 Raw264.7 macrophages with or without CQ treatment. Green, NF-κBp65; blue, DAPI. Scale bar, 20μm. **e,** Cytoplasmic and nuclear fractionation analysis of NF-κBp65 in Mcoln1-SGGFP and Mcoln1-SG1 Raw264.7 cells with or without CQ treatment by western blot. **f,** Cytoplasmic and nuclear fractionation analysis of NF-κBp65 in BMDM-M2s that had been pretreated with 1μM CsA or not for 2 hours before addition of CQ or PBS by western blot. **g,** Immunofluorescent analysis by confocal fluorescent microscopy of NF-κBp65 in IL-4 conditioned Raw264.7 macrophages that had been pretreated with 1μM CsA or not for 2 hours before addition of CQ or PBS. Green, NF-κBp65; blue, DAPI. Scale bar, 20μm. Data shown are representative of three independent experiments and error bars represent mean ± s.e.m.. *, P <0.05; **, P <0.01; ***, P <0.001; ns, not statistically significant (Student's t-test).

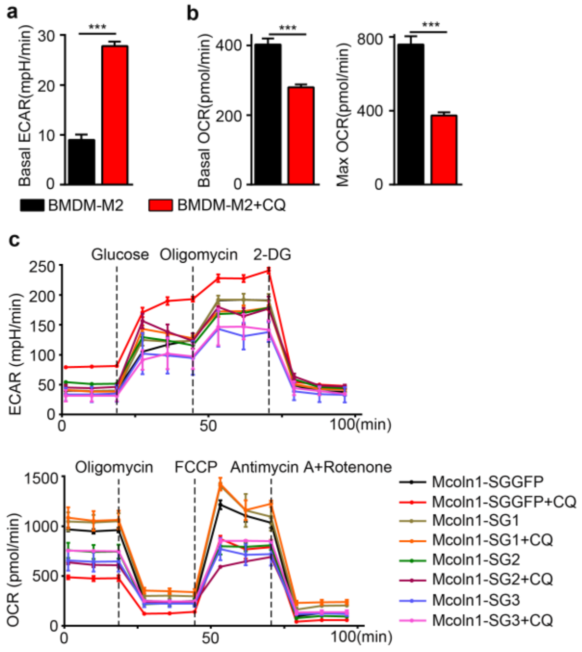

Supplementary Figure S8. Related to Figure 6.

a, Basal ECAR of BMDM-M2s with or without CQ treatment (n=3). **b,** Basal OCR and max OCR of BMDM-M2s with or without CQ treatment (n=3). **c,** The ECAR and OCR in Mcoln1-SGGFP, Mcoln1-SG1, Mcoln1-SG2 and Mcoln1-SG3 Raw264.7 macrophages with or without CQ treatment (n=3). Data shown are representative of three independent experiments and error bars represent mean ± s.e.m.. *, *P* <0.05; **, *P* <0.01; ***, *P* <0.001; ns, not statistically significant (Student's *t*-test).

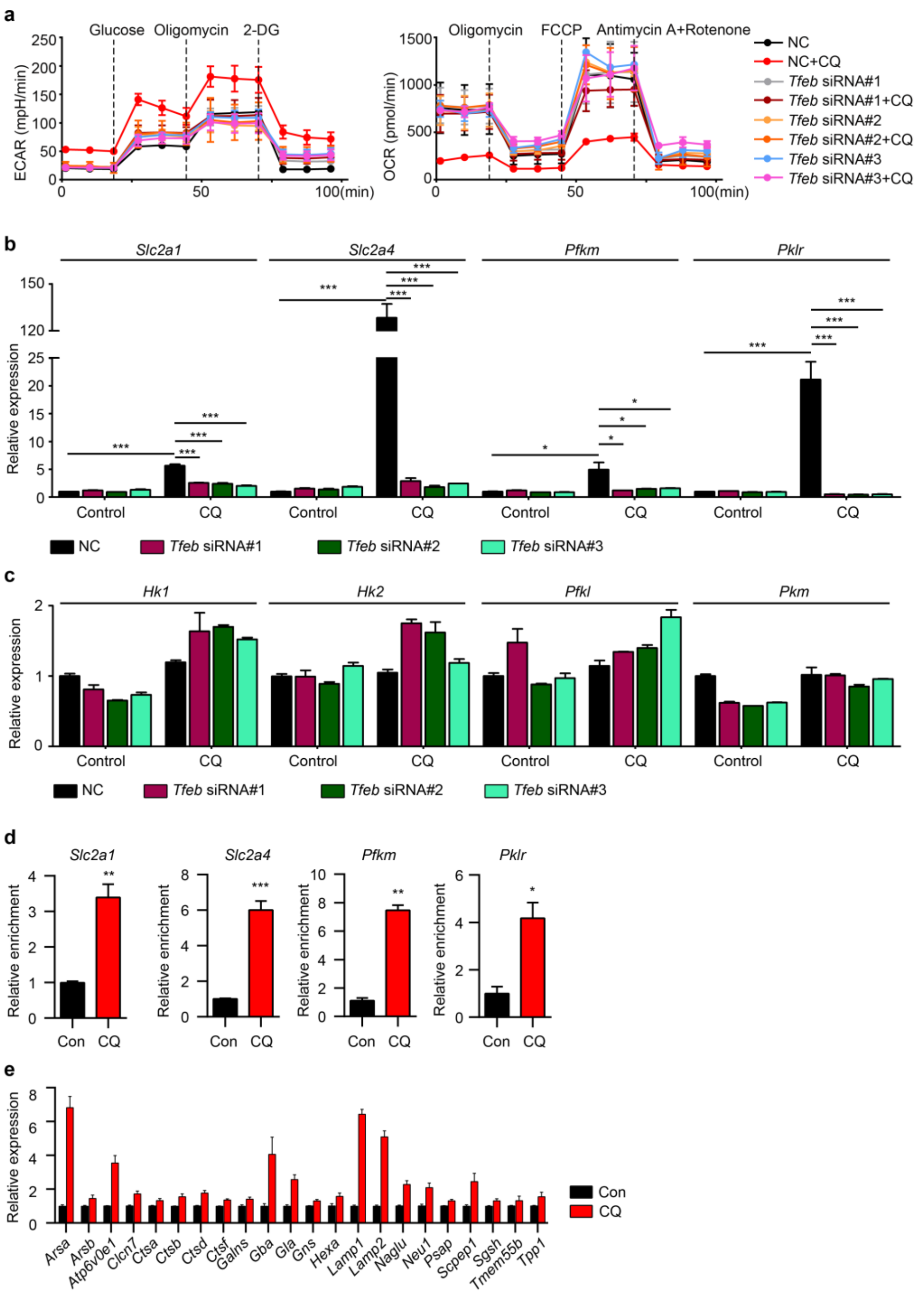

Supplementary Figure S9. Related to Figure 6.

a, The ECAR (left) and OCR (right) in control (NC), *Tfeb* siRNA#1, *Tfeb* siRNA#2 and *Tfeb* siRNA#3 transfected Raw264.7 macrophages with or without CQ treatment were analyzed (n=3). **b,** The mRNA expression of *Slc2a1*, *Slc2a4*, *Pfkm* and *Pklr* were analyzed by real-time qPCR in NC, *Tfeb* siRNA#1, *Tfeb* siRNA#2 and *Tfeb* siRNA#3

transfected Raw264.7 macrophages with or without CQ treatment. **c,** The mRNA expression of *Hk1, Hk2, Pfkl* and *Pkm* were analyzed by real-time qPCR in NC, *Tfeb* siRNA#1, *Tfeb* siRNA#2 and *Tfeb* siRNA#3 transfected Raw264.7 macrophages with or without CQ treatment. **d**, ChIP-qPCR analysis was performed with anti-TFEB and primers specific for *Slc2a1, Slc2a4, Pfkm, Pklr* in BMDM-M2s with CQ treated or not. **e,** The mRNA expression of CLEAR gene network in BMDM-M2s and CQ-treated BMDM-M2s (n=3). Data shown are representative of three independent experiments and error bars represent mean ± s.e.m.. *, P <0.05; **, P <0.01; ***, P <0.001; ns, not statistically significant (Student's t-test).

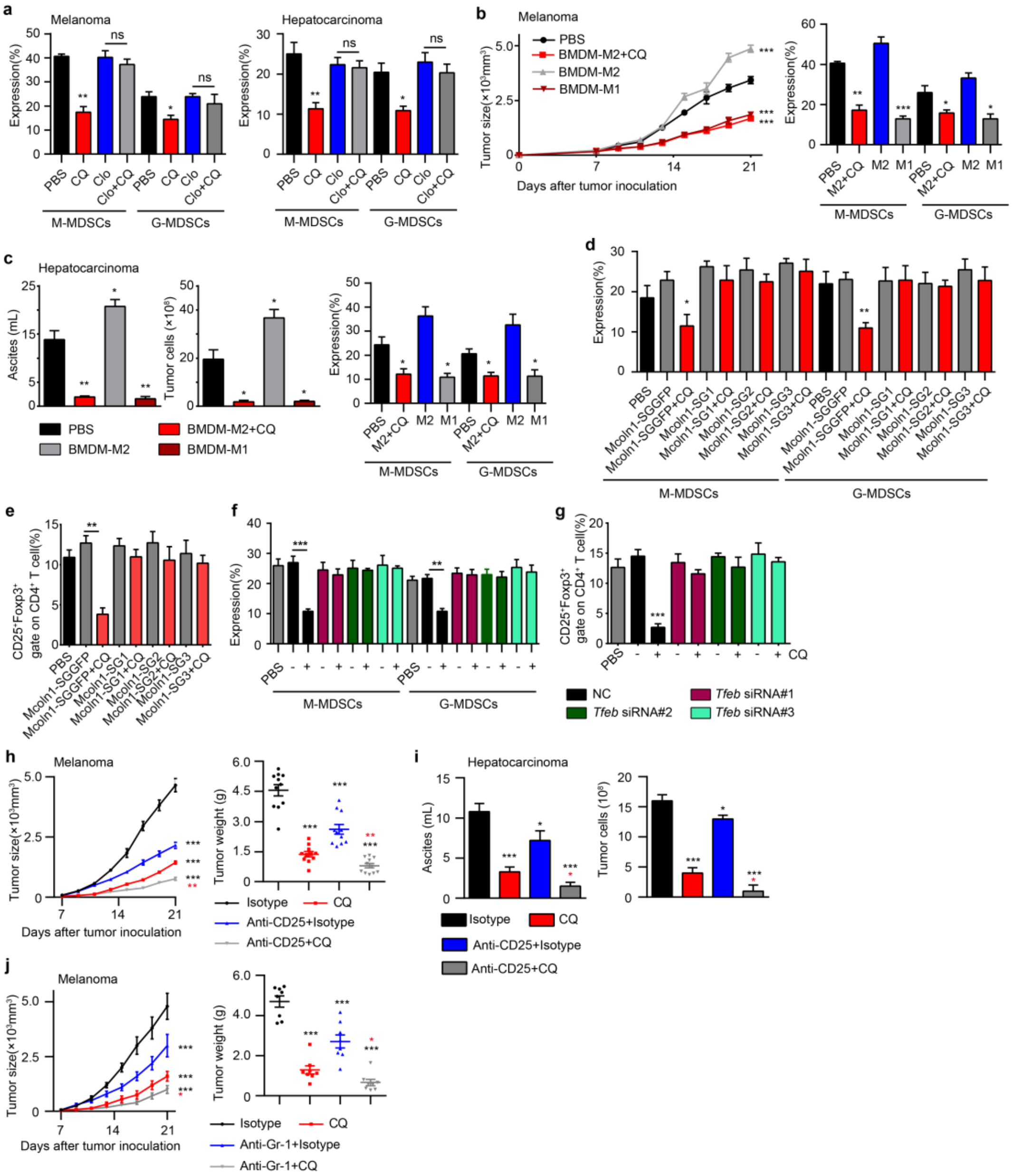

Supplementary Figure S10. Related to Figure 7.

a, B16 or H22-bearing mice that received clodronate liposomes or control treatment were given i.p. injections with PBS or CQ. Quantification of tumor associated monocytic MDSCs (M-MDSCs, $CD11b^{+}Ly6C^{hi}Ly6G^{-}$) and granulocytic MDSCs (G-MDSCs, $CD11b^{+}Ly6C^{lo}Ly6G^{+}$) numbers in melanoma (left, n=5) and hepatocarcinoma (right, n=5). **b,** C57BL/6 mice with subcutaneous B16 melanoma were treated with PBS, BMDM-M2s+CQ (CQ-conditioned BMDM-M2s), BMDM-M2s and BMDM-M1s for

two weeks. The tumor growth was followed (n=10) and the M-MDSCs and G-MDSCs number were analyzed (n=5). **c,** BALB/c mice with H22 hepatocarcinoma ascites were treated with PBS, BMDM-M2s+CQ, BMDM-M2s or BMDM-M1s cells. After one week, the mice were sacrificed to measure the tumor ascites volume (left) and the tumor cell number (right) (n=6); the M-MDSCs and G-MDSCs number were analyzed (n=5). **d,e,** The number of M-MDSCs (**d**), G-MDSCs (**d**) and Tregs (**e**) in H22 hepatocarcinoma ascites were quantified by flow cytometry in mice that received i.p. injections with PBS or cell therapy consisting of Mcoln1-SGGFP, Mcoln1-SG1, Mcoln1-SG2 or Mcoln1-SG3 Raw264.7 macrophages which had been conditioned with CQ or PBS (n=3). **f,g,** Flow cytometric quantification of M-MDSCs (**f**), G-MDSCs (**f**) and Tregs (**g**) in H22 hepatocarcinoma ascites from mice that received i.p. injections with PBS or cell therapy consisting of NC, *Tfeb* siRNA#1, #2 or #3 transfected Raw264.7 macrophages that had been pretreated with CQ or PBS (n=3). **h,** C57BL/6 mice (n=11) with subcutaneous B16 melanoma were treated with Isotype or CQ along with administration of anti-CD25 or not. The tumor growth was followed (left). After two weeks, the mice were sacrificed and the weight of the tumors was recorded (right). Black asterisk, Isotype compared with CQ, anti-CD25 and anti-CD25+CQ group; red asterisk, CQ group compared with anti-CD25+CQ group. (**i**) BALB/c mice (n=6) bearing H22 hepatocarcinoma ascites were treated with Isotype or CQ together with administration of anti-CD25 or not. Tumor ascites volume (left) and the tumor cell numbers (right) were measured after one week of treatment. Black asterisk, CQ, anti-CD25 and anti-CD25+CQ group compared with Isotype group; red asterisk, CQ group

compared with anti-CD25+CQ group. **j,** B16-bearing mice (n=8) were treated with Isotype or CQ along with administration of anti-Gr-1 or not. The tumor growth was followed (left). After two weeks, the mice were sacrificed and the weight of the tumors was recorded (right). Black asterisk, Isotype group compared with CQ, anti-Gr-1 and anti-Gr-1+CQ group; red asterisk, CQ group compared with anti-Gr-1+CQ group. Data shown are representative of three independent experiments and error bars represent mean ± s.e.m.. *, P <0.05; **, P <0.01; ***, P <0.001; ns, not statistically significant (Student's *t*-test).

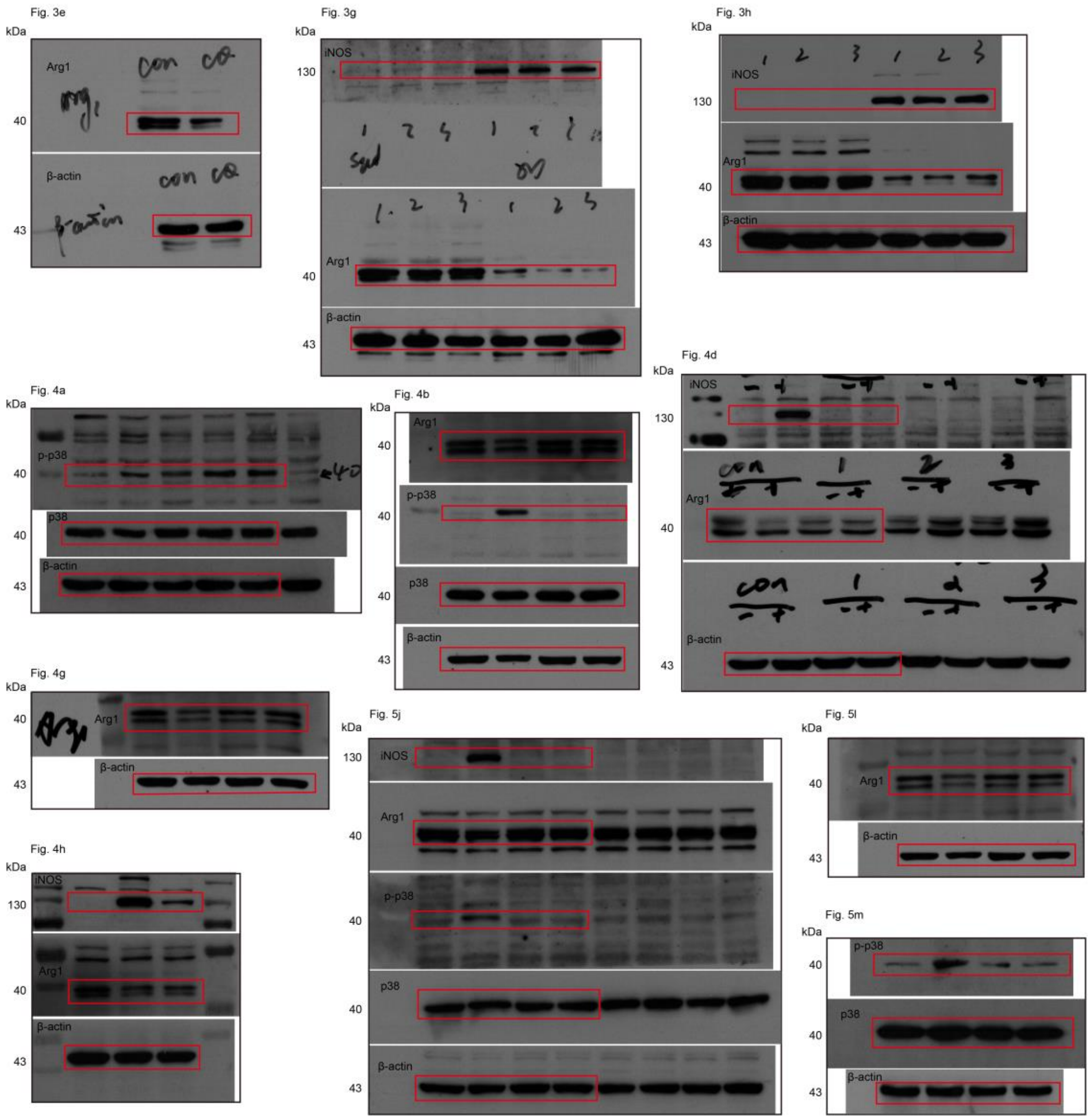

Supplementary Figure S11.

Uncropped images of western blots for Fig. 3e, 3g, 3h, 4a, 4b, 4d, 4g, 4h, 5j, 5l and 5m.

Red boxes show approximate image used for presentation.

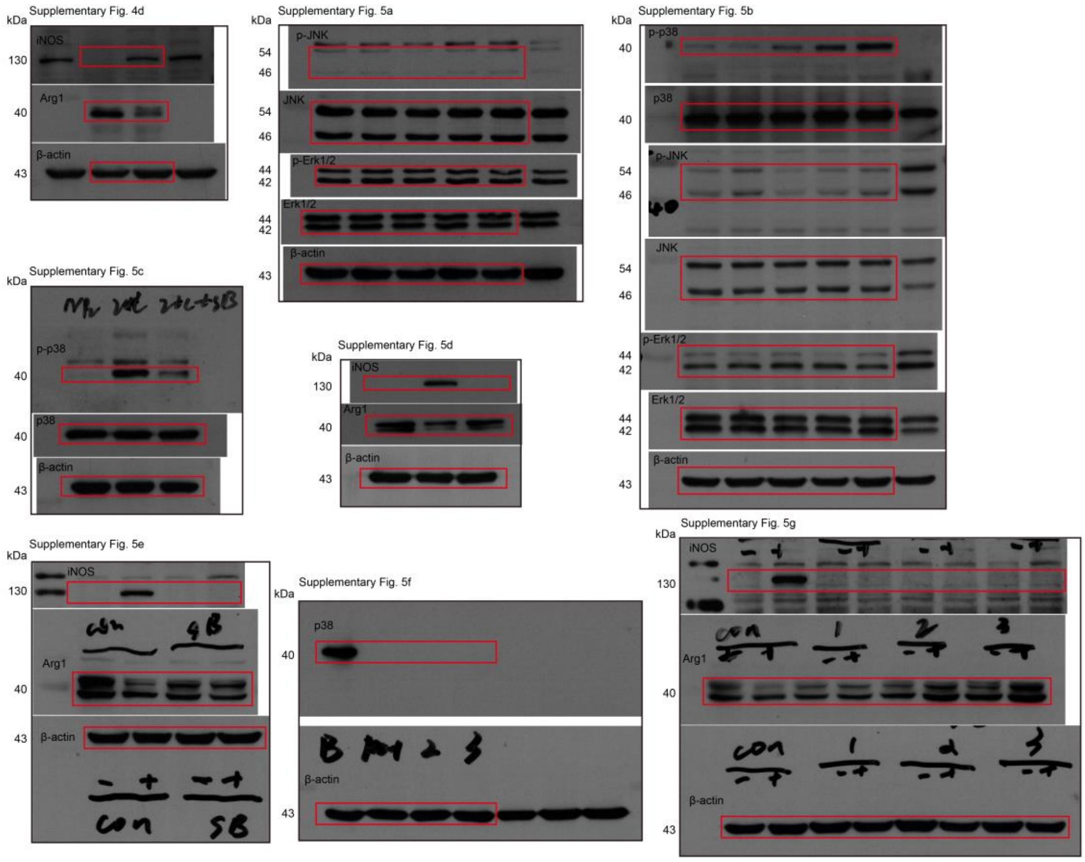

Supplementary Figure S12.

Uncropped images of western blots for Supplementary Fig. 4d, 5a, 5b, 5c, 5d, 5e, 5f and 5g. Red boxes show approximate image used for presentation.

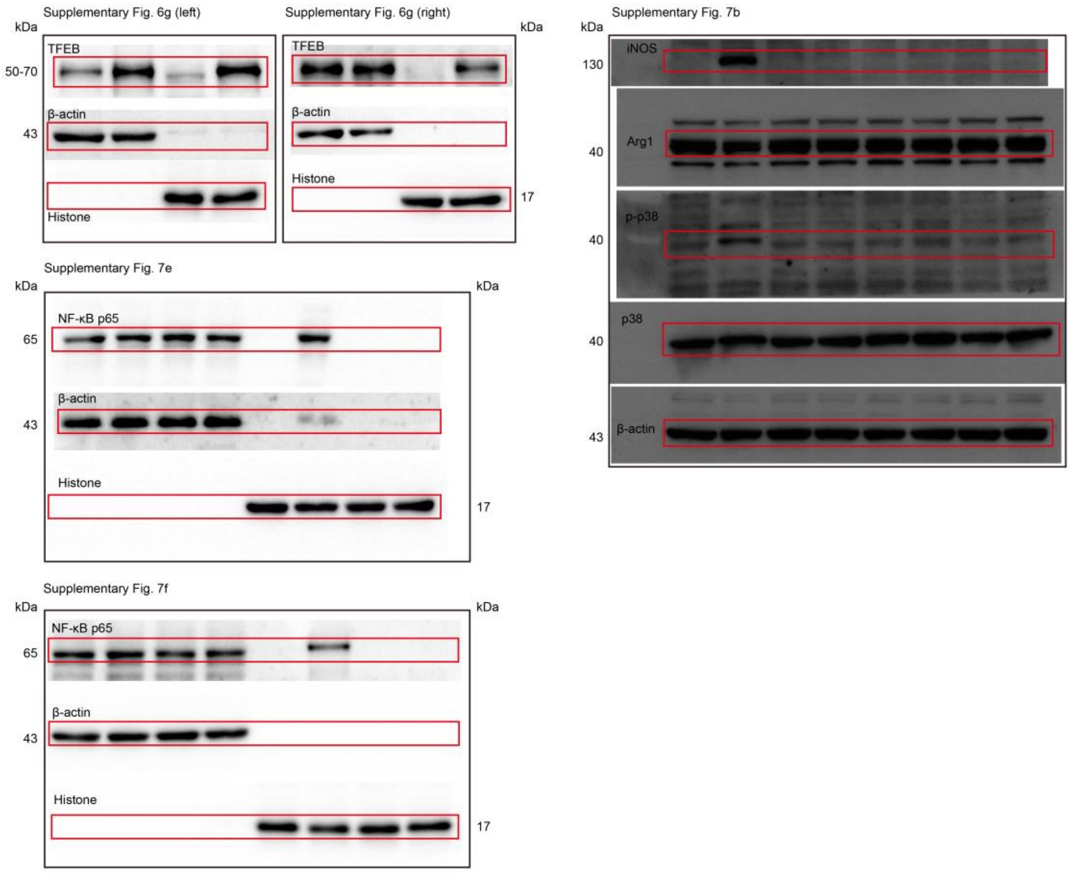

Supplementary Figure S13.

Uncropped images of western blots for Supplementary Fig. 6g, 7b, 7e and 7f. Red boxes show approximate image used for presentation.

Supplementary Table 1. Primer sequences of real-time PCR analysis

Gene name	Forward primer	Reverse primer
	Mouse	
Arg1	CAAGACAGGGCTCCTTTCAG	TGGCTTATGGTTACCCTCCC
Nos2	GATGTTGAACTATGTCCTATCTCC	GAACACCACTTTCACCAAGAC
Tnf-α	CCACGTCGTAGCAAACCAC	TTGTCCCTTGAAGAGAACCTG
IL-12p35	GGAACTACACAAGAACGAGAG	AAGTCCTCATAGATGCTACCA
IL-12p40	TGGTTTGCCATCGTTTTGCTG	ACAGGTGAGGTTCACTGTTTCT
Ifn-γ	GAGCCAGATTATCTCTTTCTACCT	GTTGTTGACCTCAAACTTGGC
Tfeb	GCTCCAACCCCGAGAAAGAG	CAGCGTGTTAGGCATCTGC
Slc2a1	GCAGTTCGGCTATAACACTGG	GCGGTGGTTCCATGTTTGATTG
Slc2a4	CTCATGGGCCTAGCCAATGC	CCCTGATGTTAGCCCTGAGTA
Hk1	AACGGCCTCCGTCAAGATG	GCCGAGATCCAGTGCAATG
Hk2	ATGATCGCCTGCTTATTCACG	CGCCTAGAAATCTCCAGAAGGG
Pfkm	CATCGCCGTGTTGACCTCT	CCCGTGAAGATACCAACTCGG
Pfkl	GGAGGCGAGAACATCAAGCC	GCACTGCCAATAATGGTGCC
Pkm	GTGGCTCGGCTGAATTTCTCT	CACCGCAACAGGACGGTAG
Pklr	GAACATTGCACGACTCAACTTC	CAGTGCGTATCTCGGGACC
Mcoln1	ACATTTGACAATAAAGCGCACAG	GGTGAGGATAACCACCACATCA
Ido1	GAGGATGCGTGACTTTGTGG	ATCAAGACTCTGGAAGATGCTG
Tgf-β1	AACAATTCCTGGCGTTACCT	GGCTGATCCCGTTGATTTCC
Il-10	CTGCCTGCTCTTACTGACTG	AAATCACTCTTCACCTGCTC
Atp6v1c1	TCTTGCCGTCTCTTCCAAGTT	AGTTCATCCGACAAGCCAACC
Arsa	AAGGTGGACTACGGTTCACAG	TACATGCCTGATCGAACTGGG
Arsb	GGCCGCTATCAGATCCATTTG	GGAGTTTTTCGTCTAGGGGAACA
Atp6v0e1	GCATACCACGGCCTTACTGT	TGATAACTCCCCGGTTAGGAC
Clcn7	CGCCAGTCTCATTCTGCACT	GAGGATCGACTTCCGGGTC
Ctsa	CAGCCCTCTTTCCGGCAATA	TTTGGGTCGTTCTGCGACTC
Ctsb	CAGGCTGGACGCAACTTCTAC	TCACCGAACGCAACCCTTC
Ctsd	GCTTCCGGTCTTTGACAACCT	CACCAAGCATTAGTTCTCCTCC
Ctsf	TGACCACCTATAACCGGACTT	TGGTGATCCCATACTGAGCTG
Galns	GCAGGCTATACCAACAAGATCG	AACCACTCGTCAAATCCATGC
Gba	GCCAGGCTCATCGGATTCTTC	CACGGGGTCAAGAGAGTCAC
Gla	TGGCGCGGACTCCTACTAT	GCCATCTGCATGAACAGTTGC
Gns	GGCATGACGCCACTGAAGAA	GGGCACATAGGCGCTAGAG
Hexa	GCTGAGGGCACGTTCTTTATC	GCGAGATGTATCCAGCAGTACG
Lamp1	CAGCACTCTTTGAGGTGAAAAAC	ACGATCTGAGAACCATTCGCA
Lamp2	ATGTGCCTCTCTCCGGTTAAA	GCAAGTACCCTTTGAATCTGTCA
Naglu	CAGCATCGAATCCTGGACCG	GCTCCCCAACTTGATGACATTG
Neu1	GGTTTGGAGTAAGGACGACGG	GGCTCCCGCTGTTTCTGAAT
Psap	TGCTGAAAGATAATGCTACGCA	GCAGGTAAGAGTCAACCACCTC
Scpep1	CTGCTGCTCCTATCGTTCTTAC	TCGGACAGTCACATAATCCCATA
Sgsh	CAGCCTTATCTTCCGTAACGC	GCCCATACATGCCATTCTGAT

Tmem55b	GCATCAGCATGTAGTCAAATGTG	GGGGCATCGGACATACTTCTT
Tpp1	GAGTCTCACTTTTGCGCTGAA	CTCCAGGGTTAGGTACTTTCCA
ActinB	CCTTCTTGGGTATGGAATCCTG	CAATGCCTGGGTACATGGTG
	Human	
IFN-γ	TCGGTAACTGACTTGAATGTCCA	TCGCTTCCCTGTTTTAGCTGC
IL-12p40	TGCCCATTGAGGTCATGGTG	CTTGGGTGGGTCAGGTTTGA
TNF-α	GAGGCCAAGCCCTGGTATG	CGGGCCGATTGATCTCAGC
Arg1	GTGGAAACTTGCATGGACAAC	AATCCTGGCACATCGGGAATC
Tgf-β1	CAATTCCTGGCGATACCTCAG	GCACAACTCCGGTGACATCAA
IL-10	GACTTTAAGGGTTACCTGGGTTG	TCACATGCGCCTTGATGTCTG
ActinB	CATGTACGTTGCTATCCAGGC	CTCCTTAATGTCACGCACGAT
